# Excess Savings Are Recession-Specific and Compensatory: Evidence From the US

**DOI:** 10.1007/s10272-022-1059-0

**Published:** 2022-08-06

**Authors:** Liviu Voinea, Prakash Loungani

**Affiliations:** International Monetary Fund, 700, 19th Street,NW ∣ HQ1-13-217, 20431 Washington, DC, USA

**Keywords:** D14, E21, E32

## Abstract

There is a consensus among academics and policymakers that the excess savings built up by households during the past couple of years are specific to the pandemic. Based on data from the past half century for the US, this article shows that savings generally increase during recessions; the pandemic is different only by the magnitude of these savings, but not by their sign. Moreover, it suggests that these excess savings are rather compensatory than precautionary, as households save more to rebuild their lost wealth.

## Higher savings during recessions

There are many ways to calculate excess savings, but it is undeniable that households stashed away piles of money during the COVID-19 pandemic (Krugman, 2021). A consensus has been growing among academics and policymakers that the excess savings built up by households since the outbreak of the coronavirus pandemic are specific to the pandemic and mainly due to the lockdowns enforced at different stages. “COVID-19 made Americans into super savers…as a result of being stuck at home” (Carpenter, 2021), “because they are not dining out or going on vacation due to the pandemic” (Bilbiie et al., 2021). “In contrast to previous economic recessions, the containment measures…saw a significant suppression of consumer spending opportunities, leading to a sizeable contraction in private consumption” (Attinasi et al., 2021). Two other explanatory factors for higher savings favoured by analysts are the massive income support measures and uncertainty (Bilbiie et al., 2021; Attinasi et al., 2021; The Economist, 2022).

* The views expressed in this paper are those of the authors and do not necessarily represent the views of the IMF, its Executive Board or IMF management.

**Liviu Voinea**, International Monetary Fund, Washington, DC, USA.

**Prakash Loungani**, International Monetary Fund, Washington, DC, USA

The main problem with the argument of excess savings being the by-product of the pandemic is the timespan of the analysis. To test whether excess savings are specific to the pandemic, we investigate the time series provided by the FRED database for the US from 1960 onwards. One way to look at it is simply by relating the quarterly data of year-on-year changes (to control for seasonality) in personal savings to the recession episodes. As seen in Figure 1, savings generally increased during recessions, except for the 1973-75 recession – which could be explained by the large inflation at that time. However, when we look at annual data, even that exception dissipates. Figure 2 shows the dynamic of annual personal savings during recessions; data for recessions are calculated as the ratio between nominal savings in the year when a recession ends to the nominal savings in the year before a recession starts. For comparison, we also indicate the percent change of nominal savings in the year before the recession and in the first year after the recession. The story told by Figure 2 is very compelling: savings increase in every recession, significantly faster than before the recession, and drop abruptly when the recession ends. Also, the deeper the recession, the higher the savings ratio – as seen in the milder numbers for the short-lived recessions of the 1990s and early 2000s, versus the larger stockpiling during the first oil shock, the global financial crisis (GFC) and the COVID-19 pandemic.

We take the analysis further, in order to control for inflation and for longer-term trends. Figure 3 presents the excess savings, calculated as the difference between the counterfactual savings (based on what the five-year average at the beginning of the recession would have predicted) and actual savings, all in real terms (at 2021 prices). The existence of excess savings can be documented for all recessions; the extent of these excess savings varies from 10%-20% above the counterfactual savings in the earlier recessions, to 60% in the GFC and 120% during the pandemic.

Figure 1

**Quarterly personal savings, year-on-year change, US, 1961-2021**

Percent change from year ago

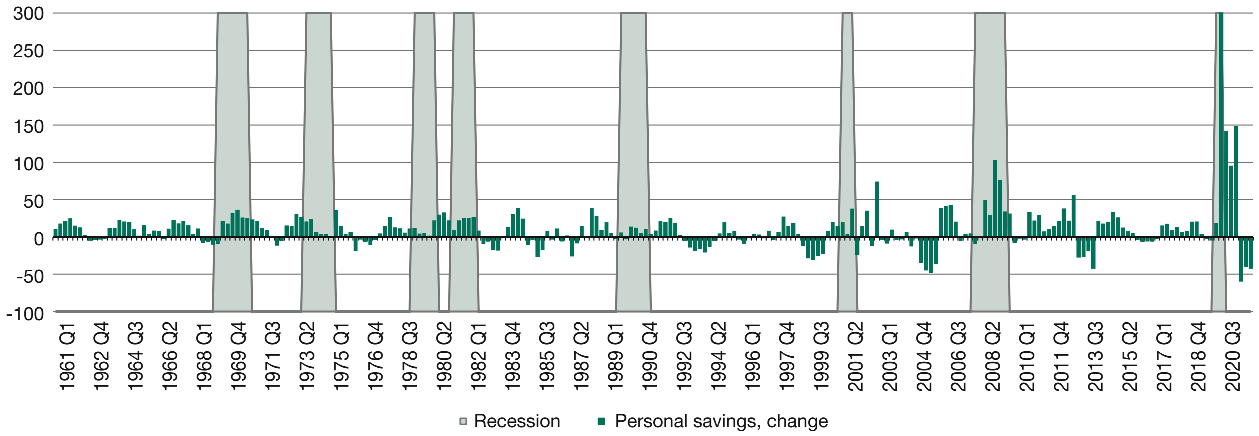

Source: Authors' calculation, based on FRED data.

## How was the pandemic-induced recession different?

Our analysis shows that excess savings are not pandemic-specific. The pandemic is different from other recessions only by the magnitude of these additional savings. The lockdown may explain part of this difference, but it is not the trigger of the excess savings phenomenon. The incapacity to access traditional retailers in the first months of the pandemic was overcome by the surge in online retail. According to the US Census Bureau, US e-commerce sales grew by a staggering 44% year-on-year and 31% quarter-on-quarter in Q2 2020 (up by more than US $50 billion compared to Q1 2020), which was the first full quarter of the lockdown, almost compensating for the loss of traditional commerce (3.6% fall in total retail sales in the same period). In fact, lockdowns had the opposite effect on consumption in the first weeks as people stockpiled goods (Baker et al., 2020). The year-on-year e-commerce sales recorded huge advances in the following three quarters (36% in Q3 2020, 31% in Q4 2020 and 39% in Q1 2021) to moderate later, but remaining positive in the recent quarters. The direct transfers are also part of the story, but only to a limited extent in the beginning; in fact, only 14% of households saved their stimulus check in the first round of payments, a share that grew to 26% in the second round and 32% in the third

Figure 2

**Nominal personal savings, annual growth rate, all recession episodes, US, 1969-2021**

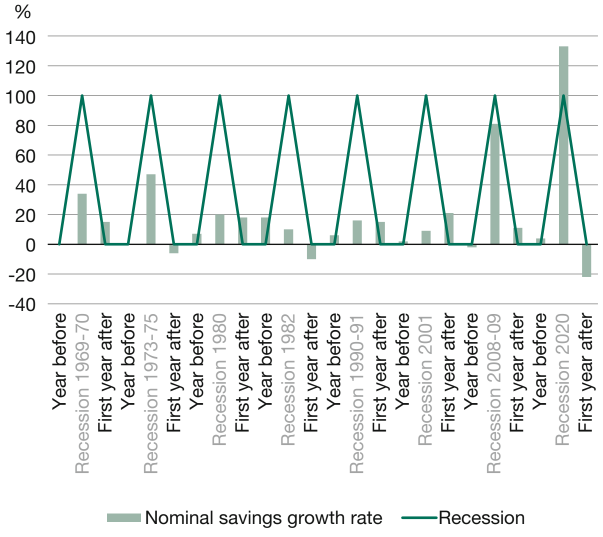

Notes: Year before: S(T-1)/S(T-2); recession: S(T)/S(T-1); first year after: S(T+1)/S(T), where T is the last year of the recession (e.g. if a recession lasts two years, T is the second year), T-1 is the last year before recession, and T+1 is the last year after recession.

Source: Authors' calculation, based on FRED data.

Figure 3

**Real excess savings, all recession episodes, US, 1969-2021**

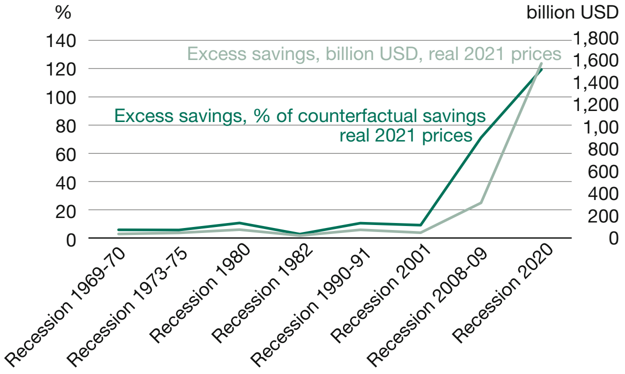

Source: Authors' calculation, based on FRED data.

Figure 4

**Personal savings rate vs real interest rate, 1982-2021**

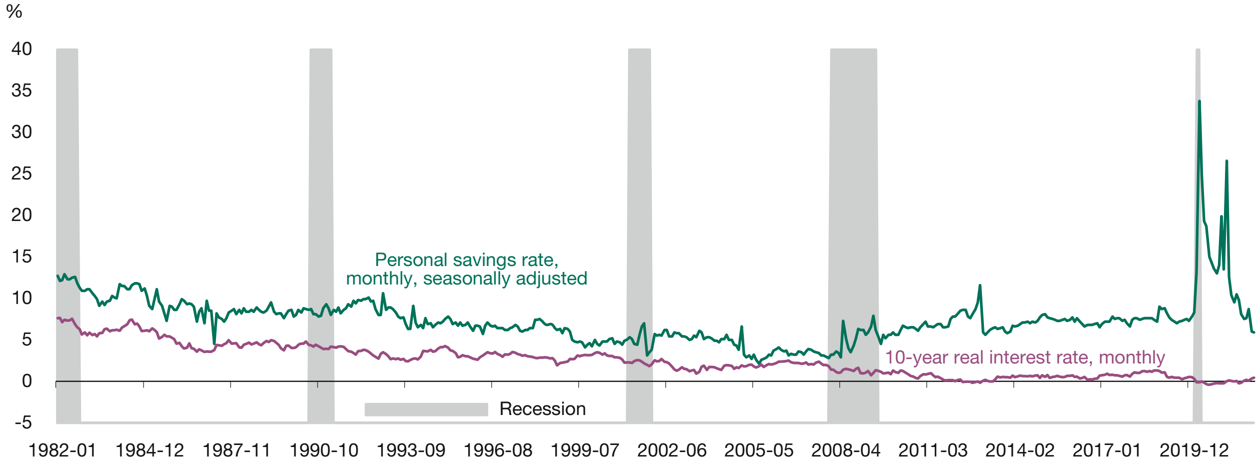

Note: FRED time series for the real interest rate starts in 1982.

Source: FRED data.

round (US Census, 2021). By the time one-third of households saved their stimulus checks, the overall savings rate was already adjusting downwards. Smith (2020), using vector autoregression models, also finds that most of the savings since March 2020 have not been driven by the direct income transfers, therefore concluding that the rest is precautionary, driven by uncertainty. Still, this time was truly different, as this was not the kind of uncertainty that can be defined by a value at risk model.

What really made the pandemic different was the Knightian, radical uncertainty (Kay and King, 2021) in its first months (How does it spread? Can it be stopped? Will we survive?), which led to much higher savings in the first quarters. As that radical uncertainty was addressed when vaccines appeared and were distributed on a large scale, the savings rate also dropped much faster than in the previous recessions. Leaving apart the radical uncertainty of a pandemic, we are left with the excess savings that characterise every recession.

## Precautionary vs compensatory savings over the business cycle

Friedman's permanent income hypothesis implies that households (dis)save if a change in income is permanent and smooth consumption if it is transitory. However, consumption smoothing requires either selling assets (buffer stock theory – Deaton, 1991) or borrowing. In a recession, liquidity constraints are more binding, affecting the capacity to borrow or sell assets to smooth consumption – hence savings should adjust downwards, if the permanent income hypothesis is true.

On the other hand, the precautionary savings argument holds that an expected fall in income would determine higher savings (Deaton, 1992; Carroll, 1994). Precautionary saving in response to labour income risk (uncertain income and employment) leads to higher savings (the income effect) and hence it is associated with the convexity of the marginal utility function (Sandmo, 1970; Kimball, 1990). One should note that the precautionary savings argument is forward-looking, as people save in anticipation of a risk that has not yet taken place, while consumption smoothing happens when that risk has already materialised.

Things get more complicated in the presence of an interest rate risk, pushing households to reduce their savings (the substitution effect), hence the simple convexity of marginal utility does not ensure that a precautionary motive for saving emerges (Rothschild and Stiglitz, 1971).

The interest rate risk refers to situations when the rate of return is negative or seen as insufficient. If the rate of return (the real interest rate) is lower than the rate of time preference, then the marginal utility of present consumption is higher than that of future consumption (as it follows from the Euler equation), and households are more willing to spend at the current time. It means that even though precautionary savings react to the perception of risk (uncertainty raises expected marginal utility of savings), they still aim to accumulate wealth (Gourinchas and Parker, 2001), which is impossible if, at the minimum, the present value is not preserved.

Data shown in Figure 4 suggest that savings tend to be counter-cyclical: They drop during economic booms and rise in recessions.

Figure 5

**Personal savings vs cumulative wage gap during the global financial crisis, US**

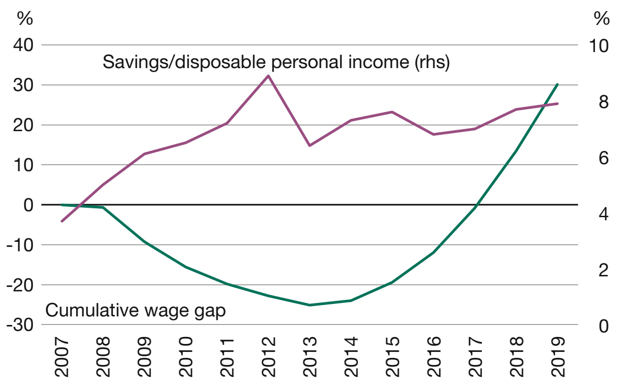

Note: The cumulative wage gap is calculated as the cumulative difference between the current wage and the last peak wage in the past, adjusted for inflation. There is a new benchmark each time there is a fall in income after a period of positive cumulative wage gap. If the cumulative wage gap is negative, another income loss is part of the same episode, not a new benchmark.

Source: Voinea (2021).

Figure 6

**Personal savings vs cumulative wage gap, during the COVID-19 pandemic, US**

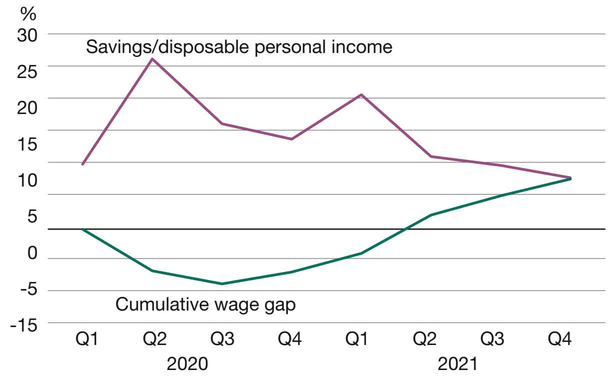

Note: The cumulative wage gap is calculated as the cumulative difference between the current wage and the last peak wage in the past, adjusted for inflation. There is a new benchmark each time there is a fall in income after a period of positive cumulative wage gap. If the cumulative wage gap is negative, another income loss is part of the same episode, not a new benchmark.

Source: Authors' calculations.

This observation is in line with some relatively recent empirical studies documenting the inverse relationship between savings and (some) recessions. Using a panel regression for 16 OECD countries, Adema and Pozzi (2015) report a similar result: When real GDP growth falls, households save a larger fraction of their disposable incomes and the opposite occurs when real GDP growth increases. Dynan (2009), Lee et al. (2010) and Mody et al. (2012) presented evidence of consumption falling and savings rising in the aftermath of the Great Recession in the US.

In Figure 4, two observations appear puzzling. First, the savings ratio has continued to rise after the GFC, as an exemption from the trend after the previous recessions. This could be explained by the impact of the quantitative easing on keeping the credit market going and supporting asset prices; in the same vein, the exceptionally high savings in the first quarters of the pandemic could have been fueled by the fact that direct transfers effectively waived off the liquidity constraints.

Second, the real interest rate is aligned with the personal savings rate in times of GDP growth, but it goes in the opposite direction during recessions (note that the FRED time series on the real interest rate only starts from 1982). Again, the exception is the post-GFC decade of the zero lower bound, when households behaved like in a recession: They continued to accumulate savings despite the low or even negative return.

These excess savings might be compensatory savings, a term first coined by Voinea (2021), indicating that households save more to compensate for a loss of wealth that has already happened (as opposed to precautionary savings, where households save more for a future risk that has not yet materialised). The idea of compensatory savings could solve the conundrum between the expected rise in savings because of the income effect and the expected drop in savings because of the substitution effect. In fact, Dynan (2009) noted that savings increase as households try to make up for capital losses, while Mody et al. (2012) found that a cut in labour income leads to an increase in the savings rate, as people try to offset their lost wealth. They referred to a loss of wealth that has already taken place, not to an uncertainty regarding the future; therefore, they were actually referring to compensatory, rather than precautionary savings.

Compensatory savings are transitory savings: They rise as income falls and drop as households gradually recover the lost wealth. As compensatory savings have their reference in the past (which is the pre-recession wealth level), they are inelastic to the dropping real interest rates. Instead, they are inversely correlated with the cumulative wage gap, which is a novel measure of the lost wealth (Voinea, 2021; Voinea and Loungani, 2021). In all US recessions since 1960, savings have been consistently inversely related to the cumulative wage gap. Figures 5 and 6 show this relationship for the GFC and the

Figure 7

**Personal savings vs cumulative wage gap, 2001-2019, Germany**

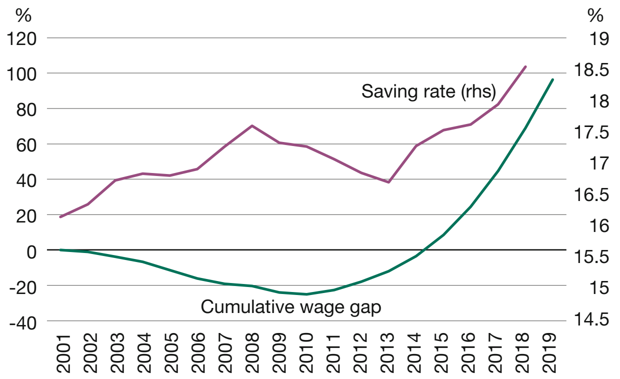

Note: The cumulative wage gap is calculated as the cumulative difference between the current wage and the last peak wage in the past, adjusted for inflation. There is a new benchmark each time there is a fall in income after a period of positive cumulative wage gap. If the cumulative wage gap is negative, another income loss is part of the same episode, not a new benchmark.

Source: Voinea (2021).

pandemic; similar results are reported for the previous US recessions by Voinea (2021). Moreover, these results are not US-specific; rather, they are recession-specific: Figure 7 shows similar findings for Germany during the past two decades.

Once the cumulative wage gap closes, the compensatory savings are either transferred into precautionary savings or into consumption. For example, after the GFC, it took eight years for the cumulative wage gap to close in the US, but even when that happened, savings did not return to their previous level, which suggested that compensatory savings were transferred into precautionary savings – an explanation which is consistent with the persistent period of low inflation in the post-GFC decade. However, as the pandemic struck, the compensatory motive kicked in again, on top of the already existent precautionary savings. As the post-pandemic cumulative wage gap has been closed much faster (by the end of 2021), the important policy question is what happens to those excess savings accumulated during the pandemic. Our educated intuition is that after the pandemic most of the excess savings will be transferred into consumption, since keeping them as precautionary savings would severely erode their value, confronted with high inflationary pressures. A similar behavior was observed during the recessions of the 1970s and 1980s which were also associated with higher inflation. The jury is still out on this, however.
